# Highly Active Carbonic Anhydrase of the Thylakoid Lumen of *Chlamydomonas reinhardtii*

**DOI:** 10.3390/plants14010055

**Published:** 2024-12-27

**Authors:** Vasily V. Terentyev, Liubov I. Trubitsina, Anna K. Shukshina, Ivan V. Trubitsin, Natalia N. Rudenko

**Affiliations:** 1 Institute of Basic Biological Problems, Federal Research Center “Pushchino Scientific Center for Biological Research of the Russian Academy of Sciences”, 142290 Pushchino, Russianataliacherry413@gmail.com (N.N.R.); 2 G.K. Skryabin Institute of Biochemistry and Physiology of Microorganisms, Federal Research Center “Pushchino Scientific Center for Biological Research of the Russian Academy of Sciences”, 142290 Pushchino, Russia; lyubov_yurevich@mail.ru (L.I.T.); tru.ivan@mail.ru (I.V.T.)

**Keywords:** *Chlamydomonas*, carbonic anhydrase, CAH3, photosystem II, recombinant protein

## Abstract

The green unicellular algae *Chlamydomonas reinhardtii* contains 12–13 carbonic anhydrases (CAs). For a long time, the two closely related α-CAs of the periplasmic membrane CAH1 and CAH2 were considered to be the CAs with the highest CO_2_ hydration activity. The recombinant protein α-CA CAH3 (rCAH3) from the thylakoid lumen obtained in the present study showed more than three times higher activity compared to CAH1 and more than 11 times higher compared to previous studies with rCAH3. Long-term sustainability of the enzyme was observed at alkaline pH (>8), with maintenance of half of its activity at 4 °C for up to 50 days. Thermostability of rCAH3 indicated the retention of the activity at 20 °C for one hour at pH 9–10 with its ~50% decrease at pH 6–7. However, the residual activity of rCAH3 after incubation at an extremely high temperature (75 °C) for 15 min led to the formation of the double-hump graph with maxima at pH 6 and 9. The enzyme demonstrated high sensitivity to ethoxyzolamide and acetazolamide at nM concentrations, to Zn^2+^ and Cu^2+^ cations at 1 mM concentrations, and L-cysteine was able to completely inhibit CA activity of rCAH3 through reduction of sulfhydryl groups. Esterase activity of rCAH3 was well detected with values comparable to those of bovine CAII, but with a maximum at pH 8 instead of pH 9, which is usual for bovine CAII. The results indicated that CAH3 may be the most active CA of *C. reinhardtii* and that its role in the photosynthetic apparatus function could have been underestimated in previous works.

## 1. Introduction

The green algae *Chlamydomonas reinhardtii* contains 12–14 carbonic anhydrases (CAs) localized in almost all compartments of the cell and belonging to three independent families of the enzyme, α, β, and γ [1,2,3]. Many of them, in cooperation with inorganic carbon (C_i_) transporters and other additional proteins, are involved in the operation of the carbon-concentrating mechanism (CCM), increasing the intracellular pool of C_i_ under low and very low CO_2_ environmental conditions [4,5,6], while the roles of other CAs are still unclear or discussed [7,8,9,10,11,12].

The first CA found in *C. reinhardtii* cells was the periplasmic α-CAH1, significantly accumulated under low CO_2_ conditions simultaneously with a high increase in total CA activity [13]. The specific CA activity of the CAH1 protein isolated with affinity chromatography was established within the range of 2000–2580 Wilbur–Anderson Units (WAU) mg^−1^ [13,14], which was near a quarter of activity obtained for bovine α-CAII (bCAII), which is one of the standard references in studies with CAs [15,16]. In 1992, another, but closely related to CAH1 (~92% similarity [17]), periplasmic α-CAH2 was isolated with ~1.5 times higher CA activity [14]. However, the maximum amount of CAH2 protein (under high CO_2_ conditions) was ~90 times less than that of the CAH1 protein [14]. Thus, it was assumed for a long time that the main CA activity of algal cells is localized in the periplasmic space and related to the highly active enzyme CAH1.

The data about the chloroplast-localized CA activity in *C. reinhardtii* cells were also obtained at the beginning of the 1990s (see in [15]); however, the fact that the main contribution to the total CA activity of the algae cell came from periplasmic CAs was not in doubt then. Around 1998, the third α-CA named CAH3 with relatively high enzymatic activity was identified and isolated by Karlsson and colleagues [18,19]. On the one hand, the immature CAH3 protein had two transport peptides clearly indicating its localization inside the thylakoids (i.e., in the lumen). On the other hand, the CA activity of CAH3 was high (~1260 WAU mg^−1^) [19] and close to that observed for CAH1 (for more details see [15]). In contrast to both periplasmic CAs (CAH1 and CAH2), the expression level of the gene encoding CAH3, as well as the content of the protein, were not strongly dependent on CO_2_ conditions of algal growth [6,12,20,21,22].

In spite of the fact that CAH3 is a lumenal protein, its role in CCM was immediately proposed through the possible CA dehydration activity (HCO_3_^−^ + H^+^ → H_2_O + CO_2_), which can result in the pass of CO_2_ formed in the reaction across the thylakoid membrane of the tubules (the thylakoids penetrating the pyrenoid) to the pyrenoid matrix [18]. However, the role of CAH3 in supporting the high photochemical activity of photosystem II (PSII) was also suggested in parallel, based on the observed results [18]. Further studies with the use of PSII-enriched membrane preparations isolated from wild type (WT) *C. reinhardtii* indeed showed the presence of a high amount of CAH3 protein in them [15,23,24,25,26]. However, the localization of CAH3 in the pyrenoid area has also been demonstrated [27]. To date, the dual role of CAH3 is still discussed by researchers, which was summarized in the recent review [15].

To study the involvement of CA activity of CAH3 in supporting the high photosynthetic function of PSII, the recombinant protein (rCAH3) was purified and used in experiments with PSII isolated from the CAH3-deficient mutant *cia3* [24]. The data showed that under C_i_-free conditions, CA activity of CAH3 could provide more than 70% stimulation of the O_2_ evolution rate of PSII from *cia3* with the addition of very low HCO_3_^−^ concentrations. The location of its acting was proposed as being in close vicinity to the PSII donor (lumenal) side. Biochemical studies showed the complete binding of added rCAH3 to the fraction of PSII-enriched membranes, as well as the requirement of the ratio of rCAH3 to PSII as 1:1 for achieving a higher stimulation effect on O_2_ evolving activity of PSII [24]. At the same time, the enzymatic properties of rCAH3 were poorly studied. There are no data on the long-term and thermal stability of the protein, the action of cations, esterase activity, etc., which are usually presented for recombinant enzymes, including CAs [28,29,30,31]. In addition, the study of the occupation of active centers by Zn^2+^ was also missing, despite the fact that the CA activity of purified rCAH3 [32] was at least 60% lower than that measured for native CAH3 [19].

The accumulation of purified rCAH3 allowed the crystallization of the protein [33], while it was possible only in the presence of a high amount of dihydrogen phosphate ions or the CA inhibitor acetazolamide. The results showed the formation of four dimers of rCAH3 per unit cell. Nevertheless, a single rCAH3 monomer was characterized by architecture similar to other α-CAs: the central core formed by the *β*-sheet, two α-helices placed at the side of the molecule, the catalytic center formed by a Zn^2+^ ion bound by three histidine residues and a water molecule in a tetrahedral geometry [33]. The rCAH3 molecule structure showed a broader cavity with the absence of the N-acetamido group, which results in structural and electrostatic changes making the protein surface more hydrophobic [33], which is probably necessary for the formation of interaction with the PSII complex.

As it was calculated from the data of the O_2_ evolving activity of PSII in *C. reinhardtii*, the flow of protons from the water-oxidizing complex to the lumen in the light is extremely high [26]. Thus, to avoid local acidification that could suppress the function of the active center of the water-oxidizing complex [24], the dehydration CA activity of CAH3 should fully cover it. In the present study, the purification of rCAH3 showed CA activity comparable to that of bCAII and long-term stability was performed. Detected values ~11 times higher indicated how much the enzymatic activity of CAH3 had been underestimated in the previous works. In addition, the main biochemical properties of the enzyme were characterized.

## 2. Results

### 2.1. rCAH3 Purification

**Figure 1:**
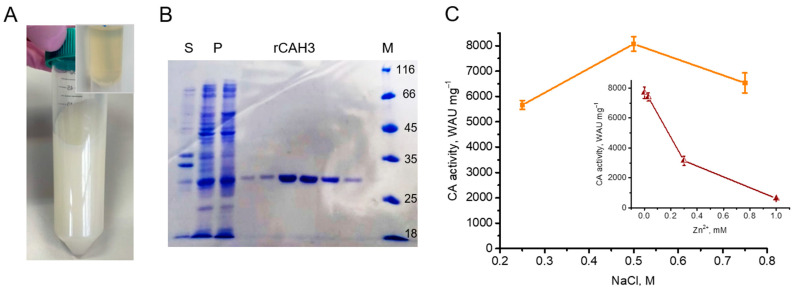
Main steps of rCAH3 purification. (**A**) A white staining suspension of *E. coli* cells with accumulated rCAH3. The inset shows an initial culture of *E. coli*. (**B**) Results of SDS-PAGE of the samples containing fractions of supernatant (S) and pellet (P) of sonicated *E. coli* cells, rCAH3 eluted from the Ni-column (rCAH3), and the markers of molecular weights (M). (**C**) CA activity of rCAH3 purified at different concentrations of NaCl in dialysis buffer, and (**insert**) the dependence of CA activity of rCAH3 on the Zn^2+^ presence in the reaction mixture.

For the isolation of rCAH3, several plasmids (pET-19mod, pET-32b(+), and pQE-30) containing the catalytic fragment of the enzyme, T5 or T7 promoters, the His_6_-tag, the cleavage sites for restriction endonucleases, antibiotic-resistance gene, etc. were constructed. At the same time, different *E. coli* strains (M15[pREP4], Origami B(DE3)) were used for transformation. The combination of pET-32b(+) with the *E. coli* Origami B(DE3) strain was similar to that used in the previous works [24,32,33]. After the addition of isopropyl-β-D-thiogalactopyranoside, the induction of the plasmid expression was observed in all variants, which was accompanied by strong white staining of *E. coli* cells suspension (Figure 1A) due to the accumulation of rCAH3 protein containing Zn^2+^ in the active site. A high accumulation of rCAH3 was supported by the appearance of a strong protein band ~30 kDa in the pellet fraction of sonicated *E. coli* cells detected by SDS-PAGE (Figure 1B). However, the accumulation of rCAH3 led to the strong aggregation of the protein with the formation of insoluble precipitate in cells. Using only the combination of pET-19mod with the *E. coli* Origami B(DE3) strain resulted in the detection of a relatively high content of rCAH3 in the soluble fraction. In this case, the following purification of the recombinant protein resulted in the collection of a product characterized by a single major band (~30 kDa) on SDS-PAGE (Figure 1B).

The protein obtained had high CO_2_ hydration activity and was obviously related to rCAH3. Measurement of its CA activity showed that it was dependent on the NaCl concentrations used in the dialysis buffer during the purification. The maximum activity (~8100 WAU mg^−1^) was detected when 0.5 M NaCl was used, and the activity was lower by ~30% (~5700 WAU mg^−1^) and by ~20% (~6500 WAU mg^−1^) if 0.25 M and 0.75 M NaCl were used, respectively (Figure 1C). At the same time, the portion of aggregated protein during this step of the purification, forming an insoluble precipitate, was also dependent on the NaCl concentration used. The solubility of the protein was lowest at 0.25 M NaCl, at 0.5 M NaCl about half of a rCAH3 portion was in the soluble fraction, and at 0.75 M NaCl almost all protein was detected in the soluble fraction (i.e., rCAH3 solubility = 0.25 < 0.50 < 0.75 M NaCl).

Thus, the maximum CA activity of purified rCAH3 was close (~87%) to that observed previously for bCAII (~9333 WAU mg^−1^ [16]), which is often used as a standard in studies with CAs. In addition, it was ~11 times higher than that observed for rCAH3 in the previous study [32]. To verify the correctness of CA activity measurements, a commercial bCAII (Sigma-Aldrich, St. Louis, MO, USA) was also used as a sample. The results showed that the value of the CO_2_ hydration activity of bCAII was equal to ~11,200 WAU mg^−1^, which was ~20% higher than that observed previously [16]. However, even in this case the CA activity of rCAH3 measured in parallel was relatively high (~75%).

One of the main interests was the occupation of the active centers of rCAH3 by Zn^2+^ atoms. This is because no addition of Zn^2+^-containing salts was applied during *E. coli* growth in contrast to the previous work where 0.5 mM ZnSO_4_ was added [24]. As seen in the insert in Figure 1C, the addition of Zn^2+^ strongly decreased the CA activity of rCAH3 with an achievement of 50% at ~0.255 mM ZnSO_4_. This indicated the occupation of all active centers of rCAH3 by Zn^2+^ atoms [31].

### 2.2. SDS-PAGE and Western-Blot Analysis of rCAH3

**Figure 2:**
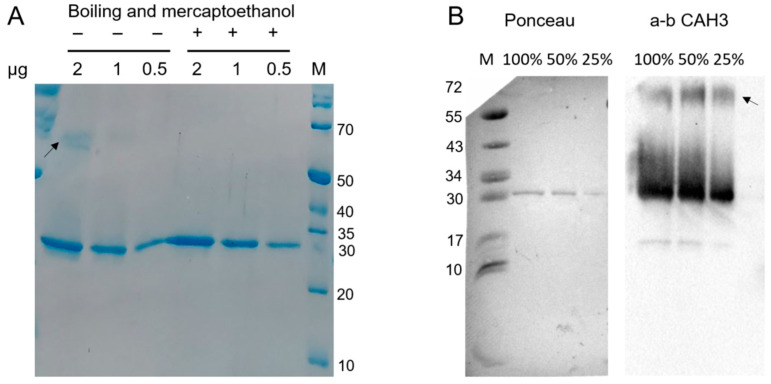
Results of SDS-PAGE (**A**) and Western-blot analysis using the primary antibodies against CAH3 (**B**) obtained for purified rCAH3 protein. The samples were loaded in terms of 2 (100%), 1 (50%), and 0.5 (25%) µg of protein per track, as indicated on top. The exposure time for Western-blot detection was 148 s. The arrows indicate the band related to a possible dimer. Staining of the membrane with Ponceau made it possible to visualize the marker bands and the major band of rCAH3.

The study of purified rCAH3 protein with SDS-PAGE besides the main major band also showed the presence of a relatively weak and diffuse band in the area of a possible dimer of rCAH3 if the samples during preparation were not boiled and β-mercaptoethanol was not added (Figure 2A). Boiling of the samples in the presence of β-mercaptoethanol led to the disappearance of this band. However, the use of the primary antibodies against CAH3 and Western blotting with long exposure time during signal accumulation made it possible to detect the presence of the weak dimer band also in samples after boiling in the presence of β-mercaptoethanol even in the case of the lowest content of rCAH3 (Figure 2B).

Such an approach also showed the presence of a highly intensive diffuse area above the main band of rCAH3, which significantly disappeared with the lowering of the rCAH3 content in the sample. Thus, the rCAH3 molecules probably can interconnect in pairs due to the hydrophobic properties of their surfaces, which is in agreement with the data observed previously during the crystallization of rCAH3 [33]. In addition, the changes in electrophoretic mobility of some molecules may occur at an increased concentration of rCAH3 in the sample.

A very weak and narrow band was also detected in the area of approximately two times lighter molecular weights (~15 kDa) compared to the main band, which can be related to a product of rCAH3 degradation. However, calculations based on the detection of the band densities indicated that this weak band contributed less than 2% to the total signal obtained for CAH3.

### 2.3. pH-Stability of rCAH3

The pH-stability of the enzymatic activity of rCAH3 was studied by the incubation of the protein at different pH values (from 10.0 to 4.0) over a time range from 10 min to 50 days and by the following measurement of CA activity with the standard approach. During the entire period of the experiments, the samples were stored at 4 °C.

**Figure 3:**
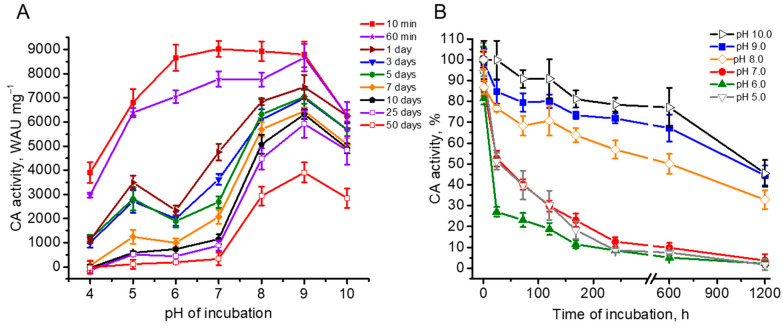
Dependence of CA activity of rCAH3 on the time of incubation at different pH values presented in absolute (**A**) and relative (**B**) units. The values obtained after 10 min of incubation were used as 100% for each pH in plot B. The incubation was performed at 4 °C during the entire time of the study. The time on the abscissa axis in (**B**) is shown in hours for better visualization and the appropriate location of the axis break. The days correspond to the following hours: 1 day is 24 h, 3 days are 72 h, 5 days are 120 h, 7 days are 168 h, 10 days are 240 h, 25 days are 600 h, and 50 days are 1200 h.

As shown in Figure 3A, under incubation of the samples during a very short time period of 10 min, CA activity showed a similar and high value in a wide range of pH from 9.0 to 6.0 with a slight maximum at pH 7, where the activity reached ~9000 WAU mg^−1^. This was ~96% of that obtained for bCAII in the previous work [16], or ~80% of that obtained for bCAII in the present study. At the same time, such short incubation of rCAH3 resulted in a decrease in the CA activity at pH 10.0, 5.0, and 4.0 by ~30%, ~24%, and ~57%, respectively.

After 60 min of incubation, a significant decrease in CA activity of rCAH3 was observed at pH 8.0, 7.0 (by ~13%), and 6.0 (by ~18%), while there were no changes at pH 10.0 and 9.0. After a day of incubation, the decrease in CA activity was detected already at pH 9.0 (by ~15%) and 5.0 (by ~51%), and the further decrease at pH 8.0 (total by ~23%), 7.0 (total by ~47%), and 6.0 (total by ~73%) but still not at pH 10.0. The most intensive loss of CA activity at pH 6.0 led to the formation of the second minor maximum at pH 5.0 in addition to the two-times higher main maximum observed at pH 9.0 (Figure 3A).

Continuous incubation of rCAH3 under different pH values revealed that at pH below 8.0, the enzyme was significantly less stable compared to that observed at alkaline pH (Figure 3). The maximum residual activity of rCAH3 was clearly detected at pH 9.0, in spite of the fact that the relative decrease in the CA activity of rCAH3 over time was the least, at pH 10.0 (Figure 3B).

Calculation of the relative decrease in CA activity of rCAH3 by 50% under incubation at different pH values showed that it was achieved after 44–46 days at pH 10.0 and 9.0 and after 25 days at pH 8.0. At pH 7.0 and 5.0, this required ~33 h, while at pH 6.0, the 50% decrease was reached already after 14 h (Figure 3B). The achievement of the 25% value of the activity was detected after ~2 days of incubation at pH 6.0 and after ~6 days at pH 7.0 and 5.0. However, at pH 10.0, 9.0, and 8.0, this value was not reached even after 50 days of incubation.

### 2.4. Thermostability of rCAH3

**Figure 4:**
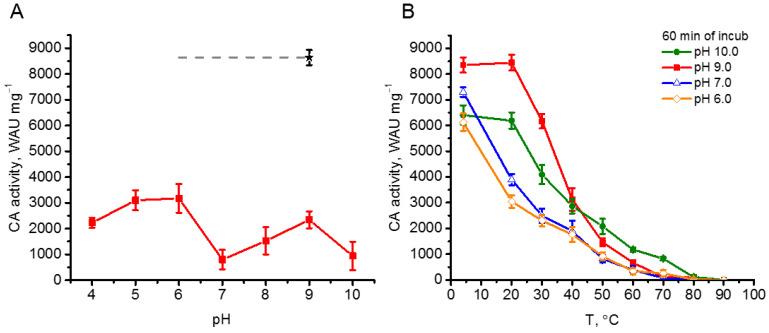
CA activity of rCAH3 under thermoinactivation. (**A**) CA activity of rCAH3 at different pH values after incubation at 75 °C for 15 min. A black star indicates the initial CA activity at pH 9 and dash line indicates it at pH 8.0, 7.0, and 6.0 according to Figure 3A. (**B**) The decrease in CA activity of the rCAH3 after incubation at different temperatures for 60 min in mixtures with different pH values.

To study the thermostability of rCAH3 the two different approaches were used. The first one was the incubation of the protein at 75 °C for 15 min at different pH values. As seen in Figure 4A, such treatment led to a significant decrease in the enzyme activity of rCAH3 (by >65% from the initial value (designated as a black star)). At the same time, the extent of the decrease was pH-dependent. A much greater suppression of CA activity of rCAH3 was detected at pH 10.0 and pH 7.0 (~9% of initial) and resulted in the formation of the double-hump graph with maxima at pH 9.0 (~27% of initial) and pH 6.0–5.0 (~37% of initial). Surprisingly, these results were contrary to those obtained previously during the study of rCAH3 pH-stability at 4 °C, when a much greater decrease in CA activity was observed after incubation at pH 6.0 (Figure 3).

The second way to study the thermostability of rCAH3 consisted of incubating the rCAH3 protein at different temperatures (4–90 °C) for 60 min. In addition, it was performed at several pH values, which provided the lowest (pH 10.0 and 7.0) and the highest (pH 9.0 and 6.0) levels of residual CA activity of rCAH3 during the first approach.

Incubation of the samples at 4 °C led to similar results observed in the study of the rCAH3 pH-stability (Figure 3), with the maximum CA activity after 60 min detected at pH 9.0 and lower values obtained at pH 10.0, 7.0, and 6.0 (Figure 4B). Incubation of rCAH3 at 20 °C did not influence its CA activity at pH 10.0 and 9.0 compared to those observed at 4 °C. However, it induced a significant decrease (by 47–50%) in the case of pH 7.0 and 6.0. Such a decrease of 50% in CA activity under pH 10.0 and 9.0 was achieved only at a temperature of ~37 °C.

After incubation at 50 °C, the residual activity of rCAH3 at pH 7.0 and 6.0 was less than 15%, while at pH 10.0 and 9.0 it was ~32% and ~18%, respectively. After incubation at 70 °C, the CA activity was detected only at pH 10.0 (~13%), and under 80 °C, the CO_2_ hydration activity of rCAH3 was almost completely lost in all variants of the study.

The results clearly showed a higher stability of rCAH3 against increased temperatures at alkaline pH, which was in good agreement with the data on the pH-stability of rCAH3 (Figure 3). At that, the algal enzyme was significantly less resistant against thermoinactivation as compared, for example, to animal bCAII, maintaining ~100% of CA activity up to 45 °C [34,35].

### 2.5. Action of Inhibitors, Cations, and Sulfhydryl-Reducing Agents

**Figure 5:**
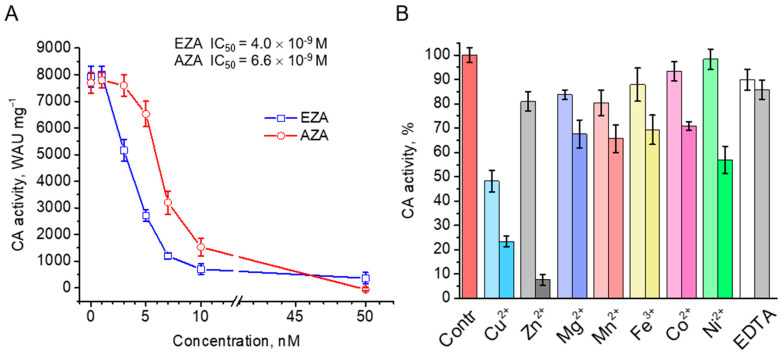
Inhibition of CA activity of rCAH3 by standard inhibitors ethoxyzolamide (EZA) and acetazolamide (AZA) with indication of the estimated IC_50_ values (**A**), and by cations of different metals and EDTA at low (0.1 mM, lighter color) and high (1 mM, darker color) concentrations added to the reaction mixture during measurements (**B**).

Purified rCAH3 was highly sensitive to the action of standard CA inhibitors, ethoxyzolamide (EZA) and acetazolamide (AZA). For both of them, almost complete inhibition of CO_2_ hydratase activity of rCAH3 was observed at concentrations close to 10 nM (Figure 5A). At the same time, the values of the half-maximal inhibitory concentration (IC_50_) for EZA and AZA were estimated as 4.0 × 10^−9^ M and 6.6 × 10^−9^ M, respectively. These results were in good agreement with those obtained previously by Mitra and colleagues [32].

The inhibitory effects of different metals were not that definitive. At 0.1 mM concentrations, almost all used metals suppressed the CO_2_ hydration activity of rCAH3 by only ~20%, with the exception of Cu^2+^, which decreased the activity by ~50% (Figure 5B). At the same time, the presence of 0.1 mM Co^2+^ and Ni^2+^ led to suppression of CA activity by only 2–7%. At 1 mM concentrations of metals, the decrease in the CO_2_ hydration activity of rCAH3 reached ~30%, with the exception of Ni^2+^ (~47%), Cu^2+^ (~77%), and Zn^2+^ (~93%). The relative decrease in inhibition for these three metals compared to the values obtained at 0.1 mM was ~42%, ~52%, and ~90%, respectively. Interestingly, in the case of such a high concentration of EDTA, which is widely used to bind metal cations, the inhibitory effect was not higher than 15% at both concentrations used, indicating strong binding of a Zn^2+^ atom with the active center of the enzyme.

The presence of the S-S bond in the CAH3 molecule between cysteines 90 and 258 is strongly important for the CA activity of the enzyme. The reduction of sulfhydryl groups, leading to disruption of the S-S bond, results in a complete loss of activity [15,33]. Usually, 10 mM concentrations of sulfhydryl-reducing chemicals are used to study their influence on the CO_2_ hydration activity of the CAs [32]. Thus, these concentrations of β-mercaptoethanol (β-ME) (~0.07%), dithiothreitol (DTT), and L-cysteine (Cys) were added to the reaction mixture during the CO_2_ hydration activity measurements of rCAH3. The results obtained indicated inhibition of CA activity to ~40% and ~30% in the cases of β-ME and DTT, respectively (Table 1), and this was consistent with previously published data [32]. However, it disagreed with data presented by Benlloch and coworkers [33], which showed the complete suppression of CA activity of rCAH3 already at ~1.5 mM DTT. It should be noted that the same additions of β-ME and DTT to bCAII also led to suppression of its CA activity to ~60%. Since bCAII does not contain an S-S bond in the molecule [36,37]. This demonstrated the presence of some inhibition action of these chemicals at such concentrations independently from the S-S bond.

The complete loss of CA activity was surprisingly observed under the addition of 10 mM Cys. However, in the work of Mitra and colleagues [32], this chemical was able to induce only a 50% decrease in activity, i.e., it was the weakest (Table 1). The suppression of CO_2_ hydration activity of rCAH3 comparable to the action of 10 mM β-ME and DTT was observed at 0.75 mM Cys, i.e., a concentration that was ~13 times lower than 10 mM. At this concentration, Cys almost did not influence the CA activity of bCAII, indicating that the effect of Cys action in the case of the rCAH3 molecule was through the S-S bond.

Usually, for detection of the inhibitory effect of DTT on CA activity, incubation at room temperature is performed [32,33], as well a neutral pH is required for the proper composition of DTT molecules for their ability to interconnect with an S-S bond. However, such incubations with 10 mM DTT and 0.75 mM Cys did not lead to a significant additional decrease in CA activity of rCAH3 (Table 1), indicating that the complete inhibitory effect of β-ME, DTT, and Cys was detected under their addition to the 3 mL thermostatic cell directly before measurements.

### 2.6. Esterase Activity

One of the specific properties known for the most α-CAs is the ability to catalyze the hydrolysis of esters, called esterase activity [38,39,40]. As shown, it correlates well with the CO_2_ hydration activity of the enzymes [41].

**Figure 6:**
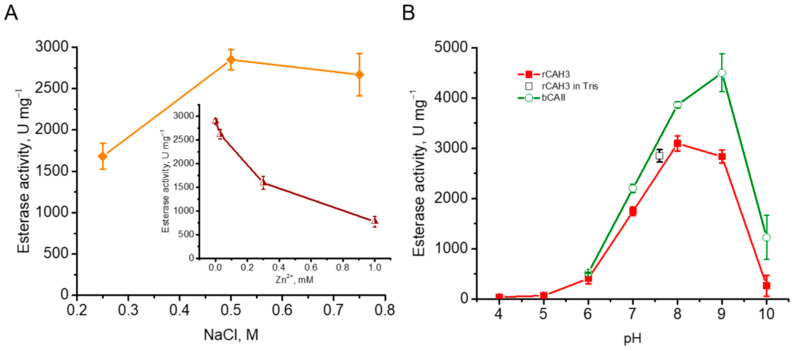
Esterase activity of rCAH3 measured in Tris buffer (pH 7.6) for samples purified at different concentrations of NaCl in the dialysis buffer (**A**), the influence of Zn^2+^ presence on rCAH3 activity in the samples obtained at 0.5 M NaCl (**insert**), and pH-dependence of the activity measured in Britton–Robinson buffer (**B**) for rCAH3 and bCAII. The black square shows the value obtained for rCAH3 in Tris buffer (pH 7.6).

Under the previously described conditions, i.e., using Tris buffer, pH 7.6 [38], the esterase activity of rCAH3 isolated at 0.5 M NaCl in the dialysis buffer was well detected in the present study and reached a value of ~2850 U mg^−1^. At that, the values obtained for rCAH3 samples purified at 0.25 and 0.75 M NaCl were different (Figure 6A). A small decrease in activity (by ~6%) was detected when 0.75 M NaCl was used and a significant decrease in activity (by ~41%) was observed in the case of 0.25 M NaCl. Moreover, the addition of Zn^2+^ led to a clear suppression of esterase activity of rCAH3 from ~2850 U mg^−1^ to ~770 U mg^−1^ (~27%) at 1 mM ZnSO_4_. Thus, these data were generally similar to those observed during the measurements of CO_2_ hydration activity of rCAH3 (Figure 1C), which, in turn, was in agreement with the suggestion about the good correlation between esterase activity and CO_2_ hydration activity of CAs [41].

Since the approach is based on the increase in absorption at 348 nm due to the hydrolysis of *p*-NPA (rather than the pH change required for the detection of CO_2_ hydration activity), it makes it possible to study the dependence of the enzymatic activity of CAs on pH. This was previously performed for bCAII and human CA B, with the detection of the maxima at pH 9.2–9.3 and 8.1–8.2, respectively [39,40]. In the present study, the same results were observed for bCAII. At pH 8.0 the activity reached values close to maximum, and the peak was at pH 9.0 (~4500 U mg^−1^) (Figure 6B). The measurements of esterase activity of rCAH3 also showed a broad peak from pH 7.6 up to pH 9.0 (activity at both points was ~92%), with the maximum at pH 8.0, where the value reached ~3100 U mg^−1^ (i.e., ~70% of that determined for bCAII). The shift of the maximum CA activity of rCAH3 to the acidic side, as compared to bCAII, was previously shown [33]. However, the maximum values observed in that study for rCAH3 and bCAII were detected at pH 6.5 and 7.0, respectively, i.e., much closer to the acidic side. This significant discrepancy can probably be explained by the specificity of the approaches used.

The comparison of the esterase activities of rCAH3 and bCAII measured at pH 7.6 independently on their pH maxima, as previously performed for bCAII [38], showed values of ~2580 U mg^−1^ and ~3200 U mg^−1^, respectively. In this case, the esterase activity of rCAH3 was ~81% of that obtained for bCAII, which is relatively high and correlates well with the high CO_2_ hydration activity of rCAH3, which was slightly lower than that of bCAII, as described above.

## 3. Discussion

CAH3 was the third α-CA found in *C. reinhardtii* by Karlsson and colleagues [18,19] in addition to the known and well-characterized CAH1 and CAH2 located in the periplasmic membrane of the algal cell [13,14,42]. The CO_2_ hydration activity detected in the precipitated fraction enriched by native CAH3 was ~1260 WAU mg^−1^ [19], which was relatively high. However, this was much lower compared to known activities of CAH1 and CAH2. Following attempts to purify the recombinant protein of CAH3 led to the obtaining of the enzyme [32] with significantly lower CA activity (by 1.7–4.2 times) compared to the native protein. Nevertheless, rCAH3 was able to stimulate up to 80% of the O_2_-evolving activity of PSII isolated from the *cia3* mutant (deficient in CAH3 in the thylakoid lumen) at extremely low bicarbonate concentrations [24]. This indicated the importance of CAH3 activity for optimal function of the photosynthetic apparatus of *C. reinhardtii* on the level of PSII.

During the production of rCAH3 in the present study, the transformation of *E. coli* strains by different plasmids containing the catalytic fragment of CAH3, including the combination similar to those used in previous studies [24,32,33], resulted in a high accumulation of rCAH3 in cells in all variants (Figure 1A,B). However, rCAH3 was prone to complete precipitation into an insoluble fraction during accumulation in cells. The only use of the pET-19mod plasmid and the *E. coli* Origami B(DE3) strain for transformation made it possible to obtain enough amount of rCAH3 in the soluble fraction. An additional requirement to obtain the maximum activity of rCAH3 was the presence of 0.5 M NaCl in the dialysis buffer (Figure 1C and Figure 6A).

The maximum CO_2_ hydration activity of purified rCAH3, in turn, was surprisingly high (up to 9000 WAU mg^−1^) and much exceeded that obtained in the previous studies by more than 11 times. The measurements with bCAII, performed in parallel, showed that the activity of rCAH3 was ~80% of that. The same high level was observed for the well-determined esterase activity of rCAH3, detected mostly in α-CAs [43,44]. In this case, the maximum value was also comparable (~70%) to that detected for bCAII in parallel measurements (Figure 6). Moreover, the CO_2_ hydration activity of rCAH3 was higher than those known for CAH1 and CAH2 by ~3.5 and ~2.7 times, respectively (for comparison see [15]). Thus, the data indicated that CAH3, located in the thylakoid lumen, probably could be the most active CA of the *C. reinhardtii* cell.

In addition, rCAH3 showed good long-term stability at alkaline pH, maintaining more than half of its CO_2_ hydration activity under incubation at 4 °C for up to 50 days (Figure 3). Similar results were observed during thermoinactivation of the enzyme, which showed higher temperatures for the ~50% decrease in its CA activity under alkaline pH, compared with those at acidic pH (Figure 4). Surprisingly, these data were in contradiction with the previously published results, which showed the place of the maximum rCAH3 activity at pH 6.5 with the decrease in values at lower pH 6.0 and higher pH 7.0 [33]. To verify the pH dependence of enzyme activity of rCAH3 produced in the present study, the esterase activity was measured at pH from 4.0 to 10.0 in comparison with that of bCAII. The results obtained for bCAII were in good agreement with the data shown previously [40]. The maximum activity in the case of rCAH3 indeed was shifted to the acidic side compared to that observed for bCAII, which was consistent with previously published data [33]. However, the maxima were detected at pH 8.0 and 9.0, respectively, for rCAH3 and bCAII, rather than at pH 6.5 and 7.0 as it was determined by Benlloch and colleagues [33]. Therefore, the long-term stability of rCAH3 correlated well with the optimal pH of its activity.

It should be noted that the esterase activity of rCAH3 decreased with pH decline and was ~56% at pH 7.0, ~13% at pH 6.0 (~35% at pH 6.5), and only ~2% at pH 6.0 (~8% at pH 5.5) from that observed at pH 8.0 (Figure 6B). This was in good agreement with the previous suggestion about the involvement of CA activity of CAH3 in supporting high PSII function at pH close to 7.0 [25,26], i.e., in conditions providing relatively high CA activity of rCAH3.

The monomer of CAH3 has a disulfide bond in its structure [15,33] in contrast to bCAII [36,37], which does not have it. The two other α-CAs of *C. reinhardtii*, CAH1 and CAH2, also have disulfide bonds, both within the monomers and between them in the dimer [45]. According to previous reports, the disruption of the disulfide bonds by sulfhydryl-reducing chemicals led to significant suppression of the CA activity of all three α-CAs of *C. reinhardtii* [32,33,46,47]. However, the observed degree of suppression in the case of rCAH3 was different between works that used the same chemicals, for example, DTT. Benlloch and colleagues [33] showed a 50% decrease in rCAH3 activity already at 0.5 mM DTT and almost complete loss of it at 1 mM DTT. In contrast, Mitra and colleagues [32] indicated a ~80% decrease in rCAH3 activity but at 10 mM DTT. The last value was close to that obtained in the present work (the ~73% decrease) (Table 1). The suppression of activity in the case of 10 mM β-ME (~0.07%) was almost the same (by ~61%).

The action of Cys was significantly different. In the present study, 10 mM Cys completely inhibited the CA activity of rCAH3, being thus the strongest inhibitor of rCAH3 CA activity found in the study. However, in the work of Mitra and colleagues [32], the 50% level of inhibition was only achieved by using Cys at the same concentration (the weakest inhibitor). The degree of CA activity suppression by Cys was similar to that observed in the presence of 10 mM DTT when the concentration of Cys was decreased to 0.75 mM (Table 1).

The presence of 0.75 mM Cys did not decrease the CA activity of bCAII, but the addition of 10 mM Cys, as well as DTT, and β-ME led, to its inhibition by 40–50%. This indicated that, besides inhibition of rCAH3 CA activity through the reduction of sulfhydryl groups, other ways of CA activity suppression also exist at such high concentrations of these chemicals.

## 4. Materials and Methods

### 4.1. Algal Growth Conditions

The *C. reinhardtii* strain CC-503 (cw92), which is a cell-wall deficient mutant [48,49] usually used as a standard wild type in photosynthetic studies, was grown at 25 °C in a 1 L bottle under aeration with 5% CO_2_ and continuous illumination with LED lamps of cool-light spectra (6500 K) with light intensity of ~100 µmol photons m^−2^ s^−1^ according to described previously [48,50].

### 4.2. Total RNA Isolation and cDNA Synthesis

Isolation of total RNA was performed with the Aurum Total RNA Mini Kit (BioRad, Hercules, CA, USA) with modifications. For cell disruption, 0.5 mL of a suspension of harvested algal cells (5000× *g*, 3 min) was frozen in liquid nitrogen and mixed with 0.7 mL of RIZol reagent (Dia-M, Moscow, Russia) containing β-mercaptoethanol immediately after thawing. The mixture was vortexed and the lysate was centrifuged for 3 min at 10,000× *g*. The supernatant was mixed with 0.7 mL of 70% ethanol and loaded onto an RNA-binding column from the Kit. Further steps were performed according to the supplied manual. In the final step, the Elution solution was loaded onto the column for 1 min and total RNA was eluted with centrifugation (10,000× *g*, 2 min). The samples of total RNA were stored at −70 °C.

Synthesis of cDNA was performed with the RevertAid First Strand cDNA Synthesis Kit (Thermo Scientific, Waltham, MA, USA) according to the supplied manual with the use of the primer complementary to 3′-end of the *cah3* gene (CTAGCACACTCGTGTCCGC). The samples of cDNA were stored at −20 °C.

### 4.3. Cloning of the cah3 Gene

The *cah3* gene of *C. reinhardtii* without the signal sequence (1–72 a.a.) was cloned into the pET-19mod, pET-32b(+), and pQE-30 plasmids. The following primers for amplification of the *cah3* gene were used: cahFe1 TTTT*AGATCT*GGAGAATCTTTATTTTCAGGGCGCAGCTTGGAACTATGGCGAAGTT and cahRe1 TAGCAC*CTCGAG*GTCCGCTCACAGCTCGTA for cloning into pET-32b(+), cahFe2 AGT*CATATG*GCAGCTTGGAACTATGGCGAAGTT and cahRe2 AGT*GGATCC*TCACAGCTCGTATTCGACCAGG for cloning into pET-19mod, and cahFe3 AGT*GGATCC*GCAGCTTGGAACTATGGCGAAGTT and cahRe3 AGT*AAGCTT*TCACAGCTCGTATTCGACCAGG for cloning into pQE-30. Cleavage sites for restriction endonucleases are presented in italics, the introduced site for TEV-protease is underscored. The cDNA was used as a template.

PCR amplification program was as follows: (1) initial denaturation at 98 °C for 30 s; (2) 35 cycles as follows: denaturation at 98 °C for 10 s, annealing at 50 °C for 30 s, elongation at 72 °C for 45 s; (3) final elongation at 72 °C for 2 min. Using the designed primers, the target PCR products were amplified and purified using a commercial kit (diaGene, Dia-M, Moscow, Russia). The correctness of amplicons was verified by sequencing.

The BamHI/HindIII-digested amplicon was cloned into pQE-30 (Qiagen, Hilden, Germany). Plasmid *pQE::cah3* was used to transform *E. coli* M15[pREP4] competent cells. The NdeI/BamHI- and Bgl II/XhoI-digested amplicons were cloned into pET-19mod and pET-32b(+), correspondingly. Plasmid *pET19::cah3* and *pET32::cah3* were used to transform *E. coli* DH5α competent cells. Transformants were selected on LB plates containing 100 µg ampicillin/mL. Insertion plasmids were obtained from the overnight culture of *E.coli* transformants. Sequences of cloned genes were verified by sequencing.

### 4.4. Recombinant CAH3 Purification

For the production of rCAH3, strain *E. coli* M15[pREP4] was transformed with *pQE::cah3*; strain *E. coli* Origami B(DE3) was transformed with *pET19::cah3* and *pET32::cah3* plasmids. All strains were grown at 37 °C with agitation at 250 rpm to a cell density of 0.6–0.8 at 600 nm. Then, 0.9 mM isopropyl-β-D-thiogalactopyranoside was added to the culture medium and the cells were incubated for 18 h at 16 °C with agitation at 100 rpm. Cells were collected by centrifugation at 4000× *g* for 30 min, suspended in 35 mL of 20 mM Tris (pH 8.0), containing 0.5 M NaCl and 1 mM imidazole (buffer 1) and disrupted by sonication. Cell debris was removed by centrifugation (90 min at 9000× *g*). The protein was purified by affinity chromatography on a HisTrap 5 mL column (GE Healthcare, Chicago, IL, USA). Cell extract was loaded onto a HisTrap column equilibrated with buffer 1, washed with six volumes of buffer 1 and then washed with six volumes of buffer 2 (20 mM Tris (pH 8.0), 0.5 M NaCl, 50 mM imidazole). Fractions containing the rCAH3 were eluted with buffer 3 (20 mM Tris (pH 8.0), 0.5 M NaCl, 300 mM imidazole). After the chromatography stage, the protein was dialyzed against 20 mM Tris (pH 9.0) buffer with 0.5 M NaCl (unless otherwise mentioned). The concentration of the protein was determined using the molar extinction at 280 nm (ε = 26.39 M^−1^ cm^−1^) calculated from the protein sequence using the Vector NTI Program (Life Technologies, Carlsbad, CA, USA).

### 4.5. SDS-PAGE and Western Blot

PAGE under denaturation conditions and Western-blot analysis was carried out as described previously [25,26,50] in 12.5% SDS-polyacrylamide gel [51] using the Mini-PROTEAN 3 Cell (Bio-Rad, Hercules, CA, USA) and the primary antibodies against CAH3 (Agrisera, Vännäs, Sweden, AS05 073). Specific modifications of the approaches are indicated in the related figures and their descriptions.

### 4.6. CO_2_ Hydration Activity Measurements

CA activity was measured at 0 °C in the 3 mL thermostatic cell as the time of the shift in pH value from 8.3 to 7.8, which was induced by the addition of cold CO_2_-saturated water to the reaction mixture, containing 25 mM Tris (pH 8.5). The saturation of water by CO_2_ was carried out on ice by passing the gas through the medium in a 100 mL glass cylinder for at least one hour and during the entire period of the measurement. The added part of CO_2_-saturated water was 40% of the total volume of the reaction mixture. CO_2_ hydration activity was expressed in Wilbur–Anderson Units (WAU) and calculated according to the equation WAU = (t_0_ − t)/t, where t_0_ and t were the times required for pH decrease in the absence and in the presence of CA in the reaction mixture. Obtained CA activities were calculated per mg of protein (WAU mg^−1^).

### 4.7. Esterase Activity Measurements

Esterase activity was measured according to the protocol described previously [38] as the acceleration of *p*-nitrophenylacetate (*p*-NPA) hydrolysis detected by the increase in absorption at 348 nm [39]. A fresh 3 mM solution of *p*-NPA was prepared every day as follows: 5.43 mg of *p*-NPA was dissolved in 0.3 mL of acetone after that water was carefully added with stirring up to the final volume of 10 mL. To obtain 1 mL of the reaction mixture in the cuvette 0.42 mL of water was mixed with 0.33 mL of *p*-NPA solution (1 mM) and 0.25 mL of 1 M buffer (25 mM). Measurement was started after 1 min of incubation and absorption at 348 nm was detected every minute for 7 min. Esterase activity was expressed in units (U), which are related to the absorption increase by 0.001 per 5 min after subtraction of values of non-enzymatic changes [38]. The measurement was conducted at room temperature. Obtained esterase activities were calculated as U per mg of protein (U mg^−1^).

### 4.8. pH-Dependence Measurements

In all pH-dependent studies, the samples of rCAH3 were diluted fourfold by 0.1 M Britton–Robinson buffer with different pH values (4.0–10.0).

### 4.9. Statistical Analysis

Statistical analysis was performed using the standard algorithms of OriginPro (2016) (OriginLab, Northampton, MA, USA). The data are presented as means ± SD.

## Figures and Tables

**Table 1:** The rCAH3 CA activity suppression in the presence of 10 mM of different sulfhydryl-reducing chemicals, β-mercaptoethanol (β-ME) (~0.07%), dithiothreitol (DTT), and L-cysteine (Cys) indicated as % of the residual activity. For DTT, the incubation at room temperature for 10 min (DTT, RT_incub_) and incubation at pH 7.0 (DTT, pH 7.0) were applied. The incubation at room temperature for 10 min in the case of Cys was performed for 0.75 mM concentrations (Cys_0.75_, RT_incub_). The values obtained in the previous studies are also indicated for comparison.

	β-ME	DTT	Cys	Cys_0.75_	DTT, RT_incub_	DTT, pH 7.0	Cys_0.75_, RT_incub_	Ref.
	% of the residual activity							
rCAH3	39.0 ± 2.8	27.2 ± 3.2	0 ± 0	28.4 ± 1.4	25.4 ± 1.8	22.7 ± 1.7	29.8 ± 1.6	this
bCAII	60.9 ± 6.4	63.4 ± 9.9	42.3 ± 7.6	97.1 ± 8.8	–	–	–	study
	% of the residual activity after RT_incub_							
rCAH3	~38	~20	~50	–	–	–	–	[32]
rCAH3		0 (reaching at ~1.5 mM DTT)						[33]

## Data Availability

The datasets generated during and/or analyzed during the current study are available from the corresponding author on reasonable request.
